# The Mexican dataset of a repetitive transcranial magnetic stimulation clinical trial on cocaine use disorder patients: SUDMEX TMS

**DOI:** 10.1038/s41597-024-03242-y

**Published:** 2024-04-22

**Authors:** Diego Angeles-Valdez, Jalil Rasgado-Toledo, Viviana Villicaña, Alan Davalos-Guzman, Cristina Almanza, Alfonso Fajardo-Valdez, Ruth Alcala-Lozano, Eduardo A. Garza-Villarreal

**Affiliations:** 1 Instituto de Neurobiología, Universidad Nacional Autónoma de México campus Juriquilla, Querétaro, Mexico; 2 University of Groningen, Department of Biomedical Sciences of Cells and Systems, Cognitive Neuroscience Center, University Medical Center Groningen, Groningen, the Netherlands; 3 Interdisciplinary Institute for Neuroscience, University of Bordeaux, CNRS UMR5297, 33000 Bordeaux, France; 4 Laboratorio de Neuromodulación, Subdirección de Investigaciones Clínicas. Instituto Nacional de Psiquiatría Ramón de la Fuente Muñiz, Mexico City, Mexico

**Keywords:** Randomized controlled trials, Magnetic resonance imaging

## Abstract

Cocaine use disorder (CUD) is a global health problem with severe consequences, leading to behavioral, cognitive, and neurobiological disturbances. While consensus on treatments is still ongoing, repetitive transcranial magnetic stimulation (rTMS) has emerged as a promising approach for medication-resistant disorders, including substance use disorders. In this context, here we present the SUDMEX-TMS, a Mexican dataset from an rTMS clinical trial involving CUD patients. This longitudinal dataset comprises 54 CUD patients (including 8 females) with data collected at five time points: baseline (T0), two weeks (T1), three months (T2), six months (T3) follow-up, and twelve months (T4) follow-up. The clinical rTMS treatment followed a double-blinded randomized clinical trial design (n = 24 sham/30 active) for 2 weeks, followed by an open-label phase. The dataset includes demographic, clinical, and cognitive measures, as well as magnetic resonance imaging (MRI) data collected at all time points, encompassing structural (T1-weighted), functional (resting-state fMRI), and multishell diffusion-weighted (DWI-HARDI) sequences. This dataset offers the opportunity to investigate the impact of rTMS on CUD participants, considering clinical, cognitive, and multimodal MRI metrics in a longitudinal framework.

## Background and Summary

Cocaine use disorder (CUD) is a worldwide public health problem with severe socio-economic consequences^1^. Clinical outcomes include attention, learning, and working memory deficit, impulsivity, and structural brain alterations^2,3^. Consequently, the pursuit of effective treatments has been a prominent focus in clinical research. Pharmacological approaches along with psychosocial therapy are currently the standard treatment with low to moderate efficacy^4^. Repetitive transcranial magnetic stimulation (rTMS) has emerged as an innovative therapeutic strategy for mitigating CUD symptoms and drug use^5–7^.

Current research is aiming towards affecting the circuits underpinning various addiction-related processes, including craving and impulsivity^8^. RTMS has the potential to activate these circuits and elicit long-term neuroplastic changes within the meso-cortico-limbic system^9^. Furthermore, the application of magnetic resonance imaging (MRI) has been instrumental in trying to find central biomarkers to measure disease severity and response to treatment.

Our dataset stems from our placebo-controlled double-blind randomized clinical trial (RCT) in which rTMS was used as an adjunct to standard treatment, referred to in this study as “treatment as usual” (TAU). The main advantage of our dataset is that we acquired longitudinal psychiatric interviews with standard clinical assessment and multimodal MRI sequences, including multi-shell diffusion-weighted imaging. For specifics on the MRI sequences obtained, please refer to the participant’s checklist available in the supplementary materials.

To date, our dataset has been used in various studies by other research teams. It has been employed to investigate both short and long-term clinical benefits of rTMS and their effects on functional connectivity^10^, to identify cognitive deficits in CUD participants through machine learning algorithms^11^, to improve diffusion MRI segmentation methods via deep learning techniques^12^, to predict clinical outcomes by examining microstructural changes^13^, and to establish a generalizable functional connectivity signature characterizing brain dysfunction in cocaine use disorder^14^. The publications and pre-prints mentioned represent just a portion of the possible applications of our data. In this release, we provide access to the complete dataset, encompassing clinical, cognitive, and MRI data, for further analysis. Previously, we have released another dataset, the SUDMEX-CONN^15^, which are independent yet acquired using the same scanner and similar sequences. Altogether, this dataset offers the opportunity to explore the longitudinal impact of rTMS as a promising adjunctive treatment for CUD and other SUDs, and to test new neuroimaging algorithms and analysis techniques. Some measurements from MRI and clinical tests have been used in different studies (See supplementary tables).

## Methods

### Participants

From a sample of n = 117 patients, 54 patients were included in the study. The reasons for the dropouts are in Supplementary Material. The study ran from May 2017 to September 2019. The study was conducted at the Clinical Research Division of the National Institute of Psychiatry in Mexico City, Mexico. The study was approved by the Ethics Committee of the Instituto Nacional de Psiquiatría “Ramón de la Fuente Muñiz” (INPRFM) (CEI/C/070/2016) and is in accordance with the latest version of the Declaration of Helsinki. The trial was registered at ClinicalTrials.gov (NCT02986438). All study participants were first interviewed to check for inclusion or exclusion criteria. Then, the patients were interviewed by the study psychiatrist, where she explained everything about the study in simple terms and followed the informed consent structure point by point. The patients were told about the absolute contraindications and possible secondary effects of the treatment, as well as how their data will be managed and disseminated. They were also told to bring a family member or another person to explain and co-sign the consent if they felt they had any doubts about it, or they could opt-out from the study without consequences at this or any point until their understanding was ensured. Patients were not paid for their participation. The patients who signed the informed consent were then included in the study. Cocaine dependence was diagnosed in CUD patients using the MINI International Neuropsychiatric Interview-Plus Spanish version 5.0.0^16^. Demographic characteristics between groups are summarized in Table 1.

**Table 1:** Demographic measures between groups.

	CUD Groups		Statistic
	Sham (n = 23)	Active (n = 30)	
	**Age**		
	33.35 ± 8.15	36.07 ± 6.82	*t*(42.61) = −1.29, *p = *0.20
	**Sex**		
*Male*	20 (86.95%)	25 (83.33%)	χ²(1) = 7.13e-32, *p* = 1
*Female*	3 (13.05%)	5(16.67%)	
	**Education in years**		
	13.40 ± 2.83	12.90 ± 3.06	*t*(49.09) = 0.60, *p* = 0.54
	**Monthly Income, MXN**		
	6591.30 ± 11386.63	5116.67 ± 6319.76	*t*(32.25) = 0.55, *p* = 0.58
	**Main substance of use**		
*Crack cocaine*	20 (86.95%)	29 (96.6%)	χ²(1) = 0.64, *p* = 0.42
*Cocaine*	3 (13.05%)	1 (3.4%)	
	**Onset age of cocaine use**		
	22.52 ± 6.76	22.77 ± 5.8	*t*(43.38) = −0.13, *p* = 0.89
	**Years of cocaine use**		
	9.59 ± 7.61	12.07 ± 7.68	*t*(47.70) = −1.17, *p* = 0.24

Continuous variables are reported as mean ± SD, and nominal as number (percentage from group): two-sample t-test and χ² was performed for each variable; no variables, n.a.: not applicable; CUD: cocaine use disorder.

### Experimental design

**Fig. 1:**
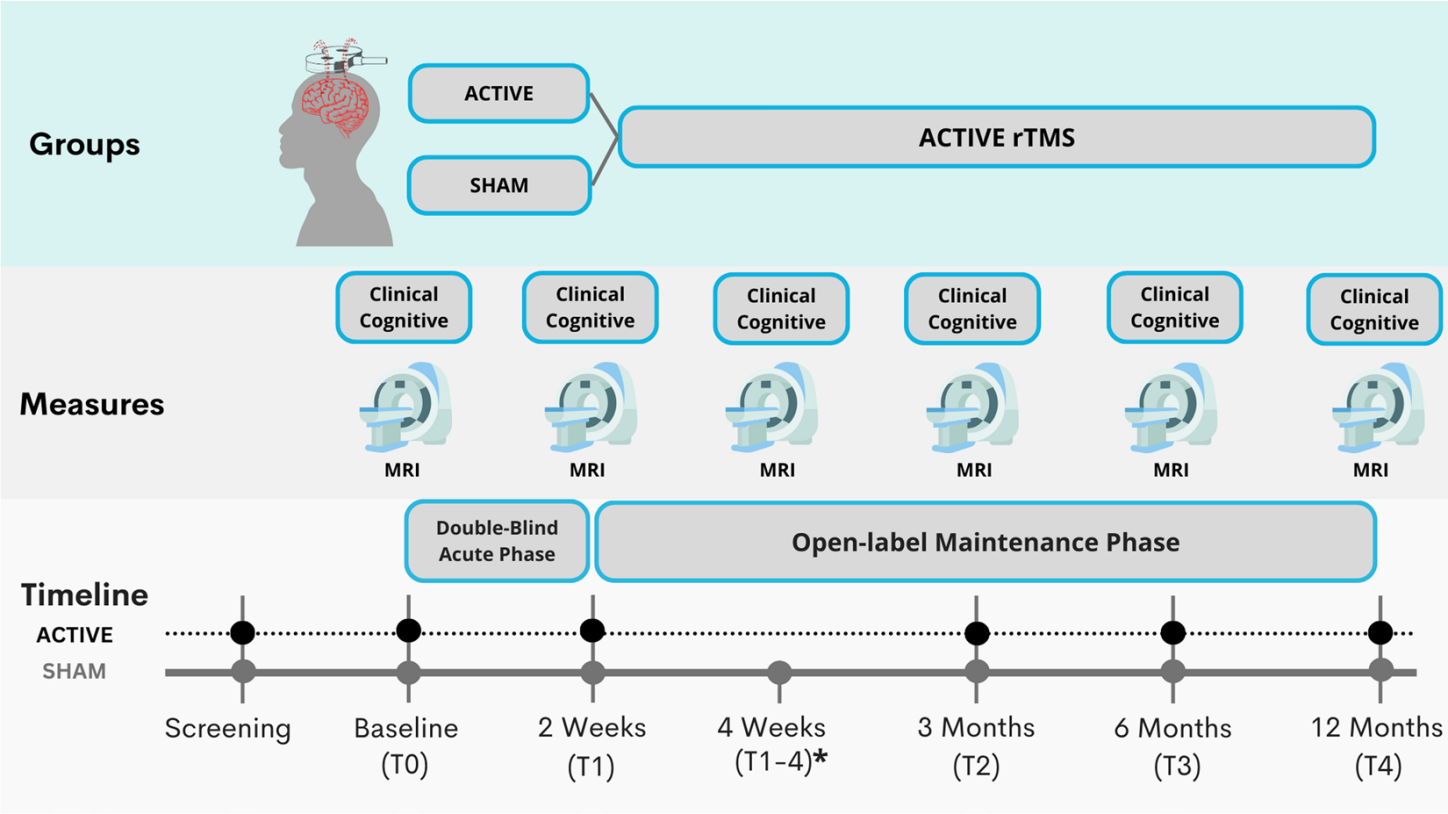
SUDMEX-TMS experimental design. Clinical and MRI data were collected at the time of baseline (T0), at two weeks (T1), three months (T2), six months (T3) and 12 months (T4); * Patients who received the sham treatment and then received active treatment (T1–4).

The study consisted of four stages: 1) a screening interview to confirm substance use disorder (SUD) diagnosis by a trained psychiatrist; 2) participants underwent a full clinical evaluation and initial MRI acquisition at baseline or Time 0 (T0); 3) an Acute stage (RCT) where patients underwent regularly scheduled sessions (Active or Sham rTMS) for 10 days over 2 weeks, after which they underwent clinical evaluation and MRI acquisition called Time 1 or T1. The blinding was opened, and only the patients in the sham group were invited to initiate active rTMS treatment for 2 weeks, after which they underwent another clinical evaluation and MRI acquisition called Time 1–4 or T1-4 (4 as in 4 weeks time). 4) Finally, after active rTMS treatment, all patients went into the open-label maintenance phase, and they underwent clinical evaluation and MRI acquisition at three months (Time 2 or T2), six months (Time 3 or T3) and 12 months (Time 4 or T4). The study design is detailed in Fig. 1.

### Study dropout

Out of the 54 recruited patients (T0), 30 were randomly assigned to active treatment, and 24 to sham rTMS. Five patients in the active rTMS group and four in the sham group discontinued the study, resulting in 24 patients completing the double-blind acute phase (2 weeks) in the active group and 20 in the sham group. Following this phase, 14 patients from the sham group chose to receive 2 weeks of acute rTMS therapy (2 weeks) after the sham phase. Participants dropouts were as follows: 1) 20 patients underwent 3 months of twice-weekly rTMS sessions, with 15 initially assigned to the active group and 5 to the sham group (T2); 2) among the study participants, 15 patients (n = 10 active, 5 sham), successfully completed 6 months of rTMS sessions (T3); and 3) 7 patients (n = 4 active, 3 sham), successfully completed 12 months of bi-weekly rTMS sessions (T4). Due to significant attrition at T1 (2 weeks), when the study was only about 30% complete, we extended the maintenance phase to 6 months instead of 12 months for new participants after obtaining approval from the ethics committee. Importantly, no adverse effects related to rTMS treatment were reported by patients who discontinued participation at any stage. None of the patients who discontinued treatment at any point reported adverse effects from rTMS.

### Study timeline

At Visit 1, the patients arrived for a clinical screening interview to confirm they met the criteria. At Visit 2, enrolled patients underwent a full clinical assessment (Time 0 or T0). Initial MRI scanning occurred at Visit 3 (Baseline or MRI-T0). The clinical interview preceded MRI acquisition and always occurred within 3 days. Following MRI acquisition, we initiated the double-blind rTMS/sham acute phase (see below). Patients underwent regularly scheduled sessions (Active or Sham rTMS) for 10 days over 2 weeks. At the conclusion of 2 weeks (Visit 4; T1), they underwent clinical assessment and repeated MRI scanning, marking the end of the acute phase and the start of the open-label maintenance phase. The blind (Active vs. Sham) was decoded for each participant at the end of their acute phase. Patients assigned to Active rTMS entered the maintenance phase directly after T1. Patients assigned to Sham rTMS were given the choice to leave the study or continue with active open-label rTMS for compassionate use. Patients assigned to the Sham group who agreed to continue, received 2-weeks (10 days) acute treatment before continuing to the maintenance phase. The maintenance phase was initially designed to include 2 weekly rTMS sessions and clinical assessments and MRI scans at 3 months, 6 months and 12 months. However, the maintenance phase was subsequently changed to 3 months for new enrollments.

### Magnetic resonance imaging acquisition

MRI sequences were acquired using a Philips Ingenia 3T MR system (Philips Healthcare, Best, The Netherlands, and Boston, MA, USA), with a 32-channel dS Head coil. The order of the sequences was the following for the single session: 1) resting state (rs-fMRI), 2) T1-weighted (T1w), and 3) High Angular Resolution Diffusion Imaging (DWI-HARDI). This order was maintained across participants. Before the MRI acquisition, the amount of alcohol in participants’ blood was measured using a breathalyzer alcohol test, and other substances were measured using a breath alcohol test and *Instant-view*^*TM*^ multi-drug urine test. The total scan time was approximately 50 min. During the study, the participants were fitted with MRI-compatible headphones and goggles (see Table 2). Anonymization of the dataset was performed using pydeface to remove facial features^17^.

**Table 2:** Summary of the acquired data for clinical measures, cognitive measures and MRI sequences.

Acquisitions		
Clinical measures	Cognitive measures	MRI sequences
• Instant-view urine test • MINI-Plus • ASI • SCID-II • SCL-R Revised • CCQ General & CCQ Now • WHODAS • BIS-11 • EHI short • HDRS • HARS • PSQI • VAS	• Berg’s Card Sorting Test • Flanker task • Go/No-go task • Letter number sequencing • Digit span backward • Iowa gambling task • Tower of london • Reading mind in the eyes	• rs-fMRI (T2*) • Structural scan (T1-weighted) • High Angular Resolution Diffusion Imaging (DWI)

Mini International Neuropsychiatric Interview - Plus; MINI- Plus, Addiction Severity Index; ASI, Structured Clinical Interview for DSM-IV Axis II Personality Disorders; SCID-II, Symptom Checklist-90-Revised; SCL-R, Cocaine Craving Questionnaire General CCQ-G and Now CCQ-N, World Health Organization Disability Assessment Schedule 2.0; WHODAS, Barratt Impulsiveness Scale v. 11; BIS-11, Edinburgh Handedness Inventory Short Form; EHI short,Hamilton Depression Rating Scale (HDRS), Hamilton Anxiety Rating Scale (HARS), Pittsburgh Sleep Quality Index (PSQI), and Cocaine Craving visual analogue scale (VAS).

### Anatomical images

T1-weighted images were acquired using a 3D FFE SENSE sequence, TR/ TE = 7/3.5 ms, FOV = 240 mm^2^, matrix = 240 × 240 mm, 180 slices, gap = 0, plane = Sagittal, voxel = 1 × 1 × 1 mm (5 participants were acquired with a voxel size = 0.75 × 0.75 × 1 mm).

### Diffusion-weighted imaging

The DWI-HARDI was a spin echo (SE) sequence, with TR/TE = 9000/127 ms, FOV = 230 mm^2^ (for 4 participants = 224mm^2^), matrix = 96 × 96 (the first 4 participants = 112 × 112), number of slices = 57 (for 4 participants = 58), gap = 0, plane = axial, voxel = 2.4 × 2.4 × 2.5 mm (the first 4 participants = 2 × 2 x 2.3 mm), directions: b0 = 8, b-value 1000 = 32 s/mm^2^, b-value 2500 = 96 s/mm^2^ (for 4 participants: 96 = b-value 3,000 s/mm^2^), with a total of 136 directions. We acquired a DWI-HARDI with an opposite direction for field mappings using a SE EPI sequence with the following parameters: TR/TE = 9000/127 ms, flip angle = 90°, matrix = 128 × 128 (the first 4 participants = 112 × 112), voxel size = 1.8 × 1.8 × 2.5 mm (for 4 participants = 2 × 2 × 2.3 mm), directions: b0 = 7, number of slices = 57 (for first 4 participants = 58), phase encoding direction = PA.

### Resting-state functional MRI

Resting-state fMRI sequences were acquired using a gradient recalled (GE) echo planar imaging (EPI) sequence with the following parameters: dummies = 5, repetition time (TR)/echo time (TE) = 2000/30.001 ms, flip angle = 75°, matrix = 80 × 80, field of view = 240mm^2^, voxel size = 3 × 3 × 3.33 mm, gap = 0, slice acquisition order = interleaved (ascending), number of slices = 36, phase encoding direction = AP. The total scan time of the rs-fMRI session was 10 min, with a total of 300 volumes acquired. All participants were instructed to keep their eyes open, and to relax while not thinking about anything in particular. We used MRI-compatible goggles (fiber optic glasses SV-7021, Avotec) to show the participants a fixation cross (white cross with black background), and we used the included eye-tracking camera to prevent participants from falling asleep during this sequence. If the participants closed their eyes for more than 10 seconds, we would wake them up using the communication through the headphones, reminding them to try to not fall asleep for the 10 minutes the sequence lasted, and we restarted the sequence over. We acquired an opposite direction sequence for field mapping, using the same GE-EPI sequence with the following parameters: TR/TE = 2000/30 ms, flip angle = 75°, matrix = 80 × 80, voxel size = 3 × 3 × 3mm, number of slices = 36, volumes = 4, phase encoding direction = PA.

### Transcranial magnetic stimulation

We performed a placebo-controlled double-blind randomized controlled trial (RCT) with parallel groups (Sham/Active) for 2 weeks (acute phase) and an open-label maintenance phase for 6 months. For the acute phase, we used a MagPro R301 Option magnetic stimulator and a figure-of-eight B65-A/P coil (MagVenture, Alpharetta, GA); for the maintenance phase, we used a MagPro R30 stimulator and a figure-of-eight MCF-B70 coil (MagVenture). The acute phase comprised 10 weekdays of 5,000 pulses per day (two sessions of 50 trains at 5 Hz, 50 pulses/train, 10 s inter-train interval, and 15 min inter-session interval). The maintenance phase comprised 3 and 6 months of 5,000 pulses per day, 2 sessions per week. The maintenance phase comprised two 5-Hz excitatory frequency (5000 pulses per day) sessions per week. The stimulation was delivered at 100% motor threshold to the left Dorso-Lateral Prefrontal Cortex (lDLPFC). The motor threshold was determined in each patient as described by Rossini *et al*.^18^. We used a vitamin E capsule as a fiducial during MRI acquisition to identify the stimulation cortical target due to a lack of a brain navigator. In all rTMS sessions, we used either the 5.5 cm anatomic Rule or the Beam F3 method. We changed to the superior Beam F3 method after the first 16 participants to improve lDLPFC localization^19^. The motor threshold was maintained at 100% in all patients. Electrodes were applied to all patient’s left temporalis muscles to simulate muscular contraction in the sham group, enhancing the sham and blinding. More information about TMS procedures is available in the supplementary material.

### Clinical measures

Patients underwent paper-based clinical tests during each session. The included assessments were: 1) Hamilton Anxiety Rating Scale (HARS), 2) Mini International Neuropsychiatric Interview - Plus (MINI-Plus), 3) Pittsburgh Sleep Quality Index (PSQI), 4) Cocaine Craving visual analog scale (VAS), 5) Addiction Severity Index (ASI), 6) Structured Clinical Interview for DSM-IV Axis II Personality Disorders (SCID-II), 7) Symptom Checklist-90-Revised (SCL-R), 8) Cocaine Craving Questionnaire General (CCQ-G) and Now (CCQ-N), 9) World Health Organization Disability Assessment Schedule 2.0 (WHODAS), 10) Barratt Impulsiveness Scale v. 11 (BIS-11), 11) Edinburgh Handedness Inventory Short Form, 12) Hamilton Depression Rating Scale (HDRS). Trained mental health psychologists and psychiatrists conducted these tests in a distraction-free environment. A summary of the acquired data is presented in Table 2. In this section, we present a selection of the metrics employed in this study. For a comprehensive description of the remaining metrics, please refer to Angeles-Valdez *et al*.^15^ and the original publications associated with each measure.

### Hamilton Anxiety Rating Scale (HARS)

The Hamilton Anxiety Rating Scale (HAM-A) assesses the global severity of anxiety and is valuable for monitoring treatment response^20^. It comprises 14 items, measuring 13 anxious signs and symptoms, with the last item evaluating the patient’s behavior during the interview. Each item is scored from 0 to 4 points, considering both intensity and frequency. The total score is the sum of each item’s score, ranging from 0 to 56 points. Optimal HAM-A score ranges are as follows: no/minimal anxiety (≤7), mild anxiety (8–14), moderate anxiety (15–23), and severe anxiety (≥24).

### Pittsburgh Sleep Quality Index (PSQI)

This instrument was developed to measure sleep quality in patients with psychiatric disorders^21^. It consists of 24 items, the assessment encompasses 7 dimensions: Subjective sleep quality, Sleep latency, Duration of sleep, Usual sleep efficiency, Sleep disturbances, Use of medication, and Daytime dysfunction. Respondents use a Likert-type scale ranging from 0 to 4. The correction involves obtaining a sleep profile for each dimension, ranging from 0 to 3, and a total score that can range from 0 to 21.

### Cocaine Craving visual analogue scale (VAS)

This instrument is designed for the subjective assessment of the participant’s current craving. The visual scale comprises a continuous line of 10 cm (including 2 decimal places). The left end represents ‘no craving,’ and the right end represents ‘the most intense craving.’ Participants are instructed to mark the intensity of their current craving by placing a cross on the scale^22^.

### Hamilton Depression Rating Scale (HDRS)

The Hamilton Rating Scale for Depression was employed to gauge the severity of depression^23^. The version we used is one of 17 items, with its content centering on depressive behavior. Vegetative, cognitive, and anxiety symptoms carry the most significant weight in the total calculation of the scale. Cutoff points for defining severity are as follows: no depression (0–7), mild depression (8–16), moderate depression (17–23), and severe depression (≥24).

### Cognitive tests

At each of the three stages, patients underwent a comprehensive neuropsychological assessment. The computer-based assessments, including Berg’s Card Sorting Test, Flanker Task, Go/No-Go task, Iowa Gambling Task, Tower of London, and Reading the Mind in the Eyes Test, were conducted using the Psychology Experiment Building Language (PEBL) version 2.0 with Spanish translation^24^. On the other hand, the Digit-Span Backward and Letter-Numbers Sequencing exams were administered on paper. All cognitive tests were administered by a licensed psychologist in a quiet setting to minimize distractions. The assessments took place after the MRI scan, with a total duration of 45 minutes per participant.

Here, we only describe some of the metrics applied, the complete descriptions of the rest of the metrics can be found in the work by Angeles-Valdez *et al*.^15^, as well as in the original publications for each measure.

## Data Records

### MRI organization

**Fig. 2:**
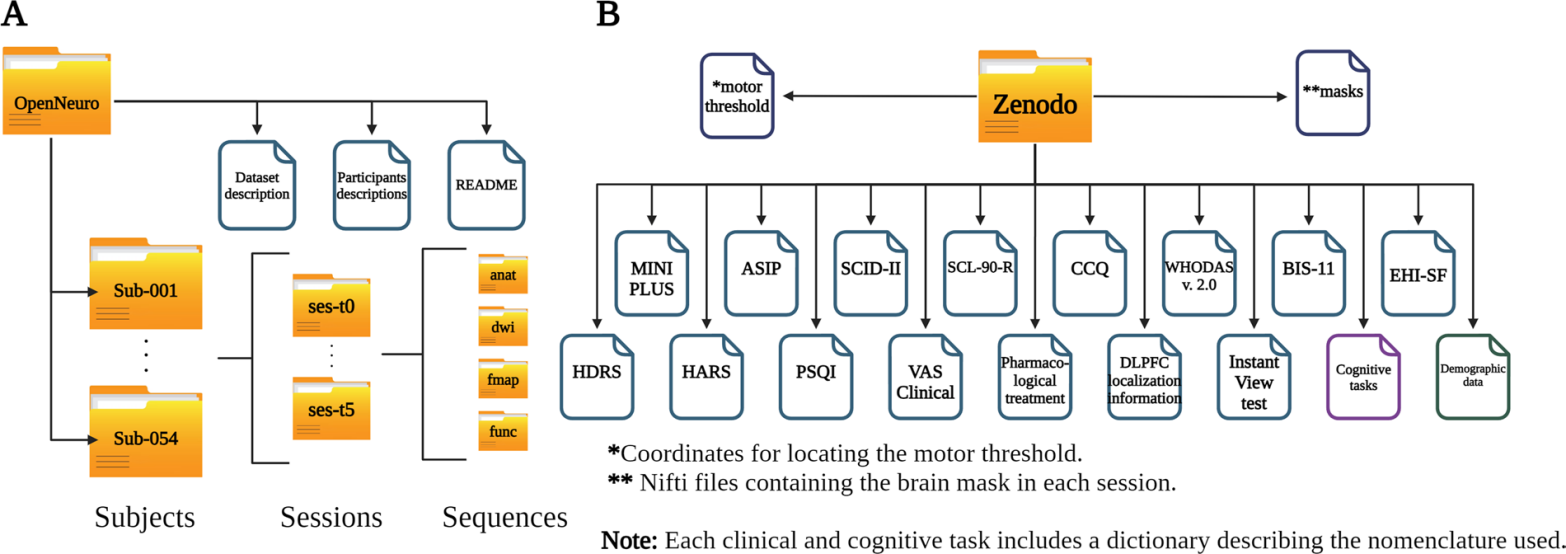
Descriptive image of the existing files in each platform. (**A**) MRI data files and BIDs organization in OpenNeuro platform, (**B**) Clinical and cognitive files and organization in Zenodo platform.

The organization of the dataset follows the Brain Imaging Data Structure (BIDS, v. 1.0.1) (https://bids-specification.readthedocs.io/), commonly used to facilitate data sharing and project unifications by folder and file name structure according to sequence modality (Fig. 2A)^25^. MRI images are shared in the Neuroimaging Informatics Technology Initiative (Nifti) format converted from Digital Imaging and Communication In Medicine (DICOM) using dcm2bids v.2.1.4^26^., along with data descriptions and metadata in JavaScript Object Notification files. The dataset is available and hosted on the OpenNeuro^27^ Data sharing platform (https://openneuro.org/datasets/ds003037/versions/2.1.0)^28^. The Brain Imaging Data Structure (BIDS) employs a hierarchical subdirectory layout. At the root resides the dataset, followed by individual session folders. Within each session, modality-specific folders organize the sequences. The MRI images are inside each folder alongside a JSON descriptor, detailing its run-specific metadata (Fig. 2A). The structure tutorial is available on the BIDS website (https://bids-specification.readthedocs.io).

### Clinical and cognitive organization

The clinical, cognitive, and demographic data are available in the Zenodo repository (10.5281/zenodo.10409461)^29^. The data set is curated and organized by test type for each experimental phase. The Zenodo repository houses each test type in an individual spreadsheet, separated by variables (columns) and each participants’ observations (rows), with individual items and total scores as variables. Additionally, a dedicated metadata sheet acts as a comprehensive glossary that defines each variable’s nomenclature, type, and level of measurement (Fig. 2B).

## Technical Validation of the MRI

### Quality Control

**Fig. 3:**
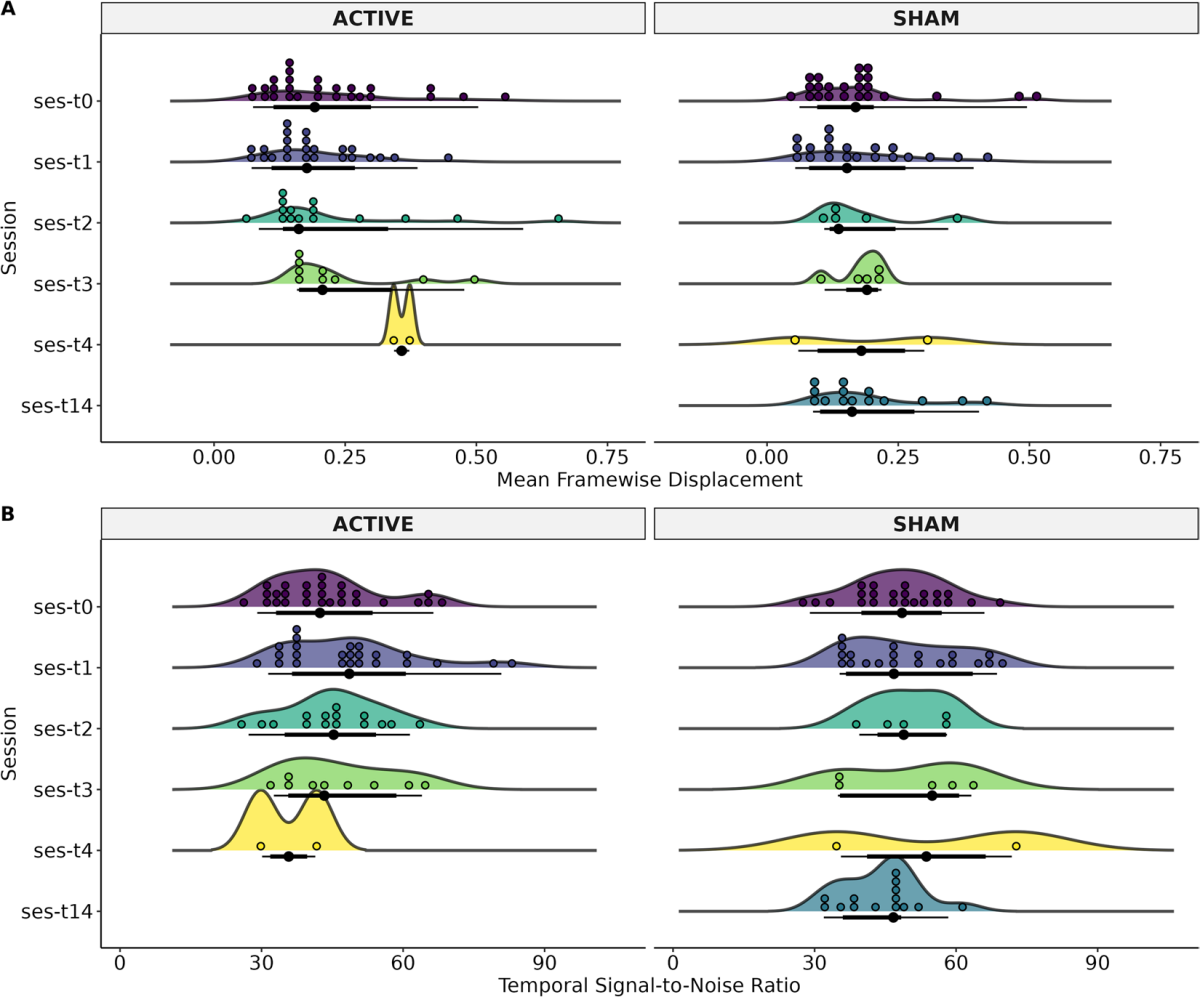
Status of the structural weighted image of each MRI-Session (ses): baseline (T0), at two weeks (T1), three months (T2), and six months (T3), and patients that had 12 months (T4). The T14 time was for patients in the Sham group who decided to continue the clinical trial with open-label rTMS. The T14 refers to 2 weeks after T1 (4 weeks after T0). (**A**) Signal-to-Noise Ratio (SNR), and (**B**) Contrast-to-Noise Ratio (CNR).

**Fig. 4:**
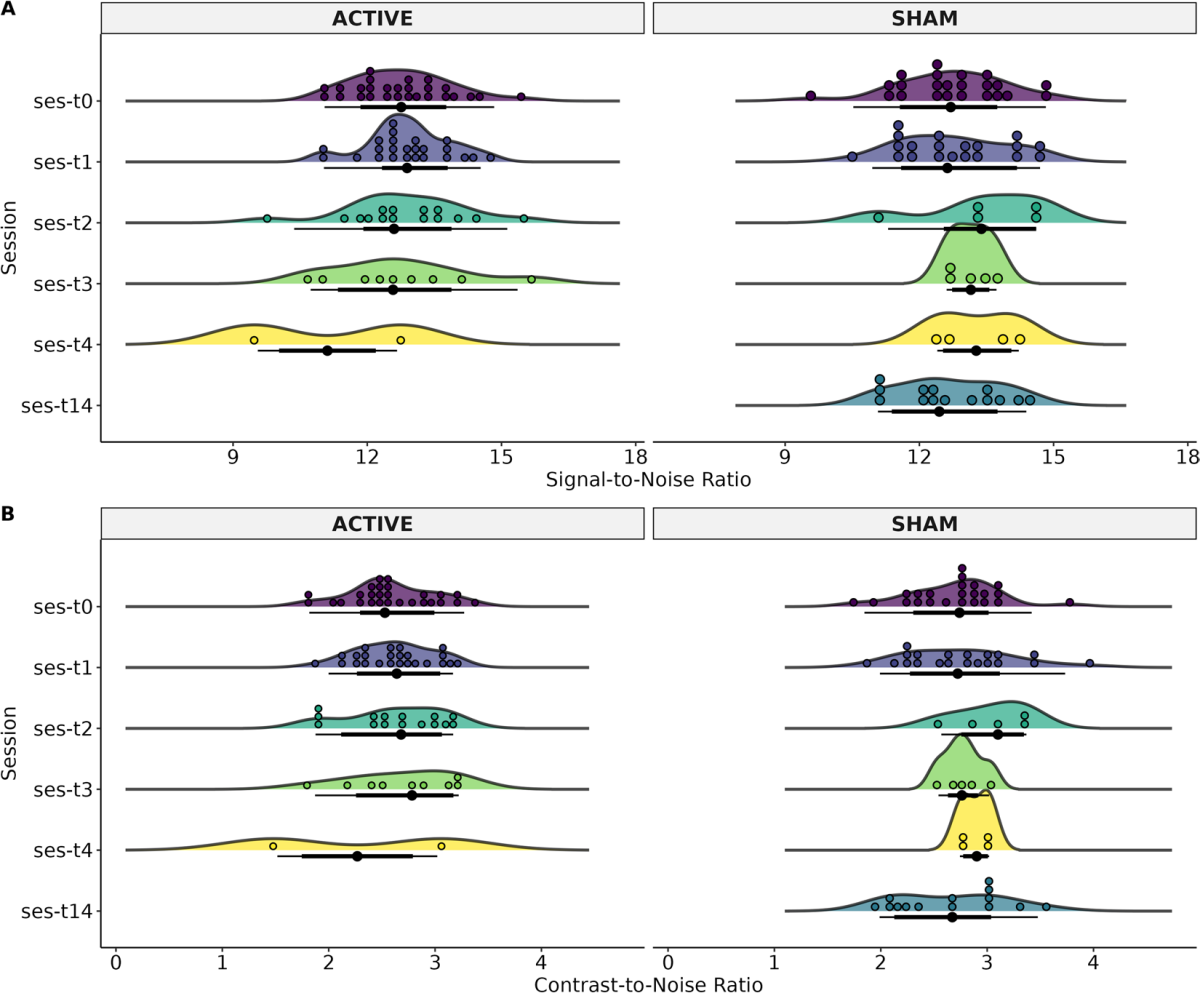
Status of the resting-state image of each MRI-Session (ses): baseline (T0), at two weeks (T1), three months (T2), and six months (T3), and patients that had 12 months (T4). The T14 time was for patients in the Sham group who decided to continue the clinical trial with open-label rTMS. The T14 refers to 2 weeks after T1 (4 weeks after T0). (**A**) Mean Framewise Displacement (FD) and, (**B**) Temporal Signal-to-Noise Ratio (tSNR).

To assess the quality control of sMRI and rsMRI sequences we used the MRIQC v.0.15 tool^30^. The extracted values for sMRI were Signal-to-Noise Ratio (SNR) and Contrast-to-Noise Ratio (CNR) (Fig. 3). SNR is related to the ratio of the mean voxel intensity of an image in contrast with the random noise intensity^31^, whereas the CNR measure is an extension of SNR that is not influenced by contrast changes^32^. For rsMRI the extracted values were Framewise Displacement (FD) and temporal Signal-to-Noise Ratio (tSNR) (Fig. 4). FD is the sum of parameters of translational and rotational realignment of head motion^32,33^, while tSNR is calculated as the division of the mean of the time series by its standard deviation, when spatial resolution increases, tSNR decreases^34^.

**Fig. 5:**
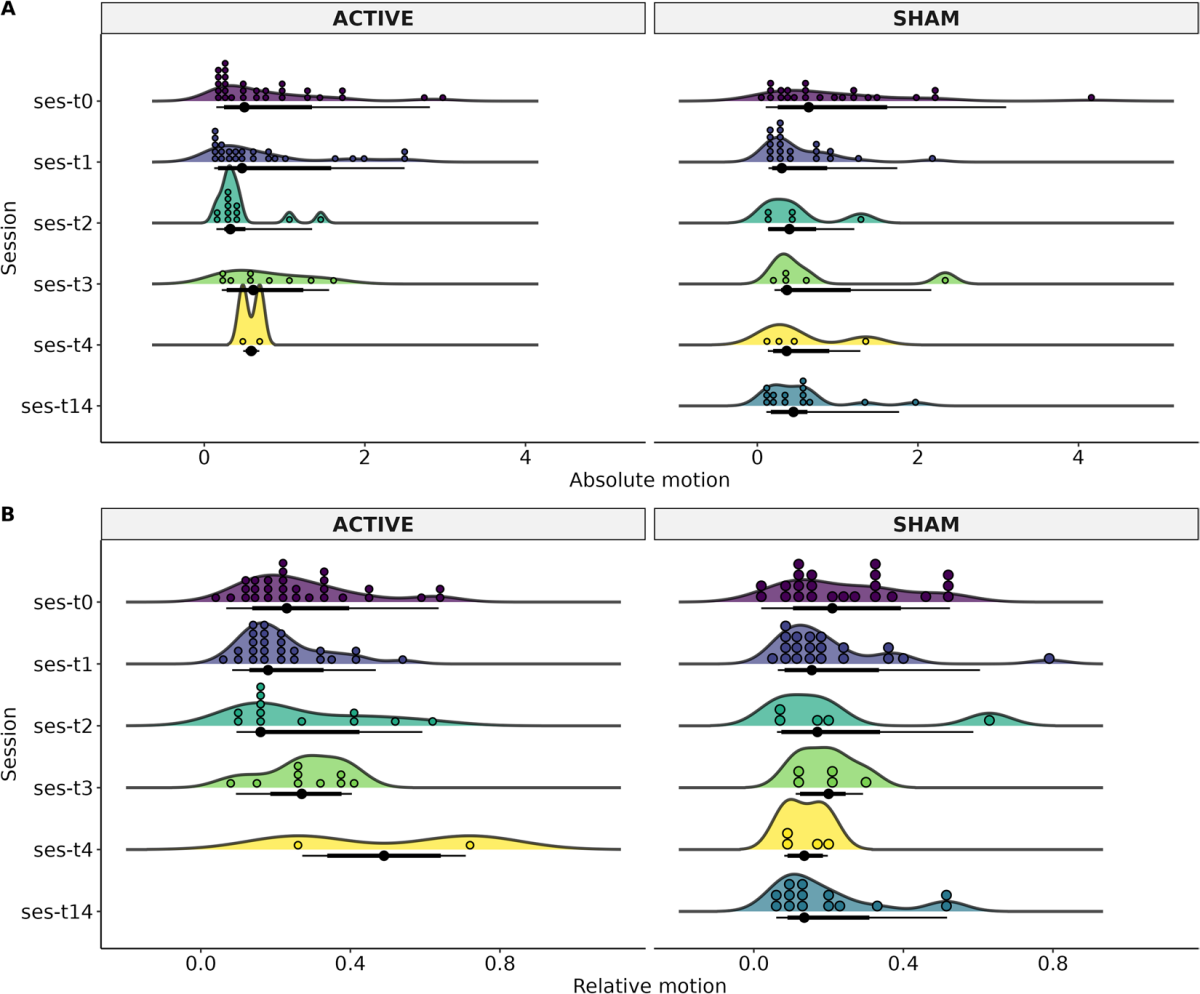
Participant motion of the diffusion-weighted image of each MRI-Session (ses): baseline (T0), at two weeks (T1), three months (T2), and six months (T3), and patients that had 12 months (T4). The T14 time was for patients in the Sham group who decided to continue the clinical trial with open-label rTMS. The T14 refers to 2 weeks after T1 (4 weeks after T0). (**A**) Absolute motion, (**B**) Relative motion.

For Diffusion-weighted images, we performed automated diffusion MRI QC (FSL EDDY_QC) to extract QC metrics sensitive and specific to artifacts^35^, at a single level. The extracted values were related to motion: absolute (motion referenced to the middle time-point) and relative (motion compared with the previous time-point). The automated QC tool relies on EDDY^36^, used to calculate the motion of dMRI data through volume-to-volume motion based on 6 parameters of the degree of freedom (Fig. 5).

## Usage Notes

The present dataset consists of cocaine use disorder patients treated using rTMS therapy followed up to 12 months and divided into a sham group and an active rTMS group for the first 2 weeks. New studies can be focused on the impact of rTMS over the active group in contrast to sham, on multimodal MRI data and/or clinical/cognitive measures. To use the present dataset in conjunction with other datasets, such as those of the ENIGMA consortium, we recommend the use of an MRI-site harmonization technique such as ComBatHarmonization^37^. Here, we provided QC information in order to facilitate data usage of which participants could be dismissed from data analysis. We recommend the use of artifact correction methods for preprocessing data due to the high motion of some subjects during scanning. The present dataset was released and peer reviewed in 2023 based on MRI OpenNeuro version 2.1.0 and Zenodo platform version 4.2.

### Supplementary information

Supplementary material

## Data Availability

The MRI dataset can be found in https://openneuro.org/datasets/ds003037/versions/2.1.0^28^. Please download the latest available version as there may be updates. Clinical and cognitive data are available in Zenodo 10.5281/zenodo.10409461^29^. No custom code was used in this work.
